# Internal control beliefs and reference frame concurrently impact early performance monitoring ERPs

**DOI:** 10.3758/s13415-018-0604-6

**Published:** 2018-05-14

**Authors:** Daniela M. Pfabigan, Anna M. Wucherer, Claus Lamm

**Affiliations:** 1 Social, Cognitive and Affective Neuroscience Unit, Department of Basic Psychological Research and Research Methods, Faculty of Psychology, University of Vienna, Liebiggasse 5, 1010 Vienna, Austria; 2 School of Psychological and Cognitive Sciences, PKU-IDG/McGovern Institute for Brain Research, Beijing Key Laboratory of Behavior and Mental Health, Peking University, 52 Haidian Road, Beijing, 100871 China

**Keywords:** Performance monitoring, Reference frame, Internal control beliefs, FRN, P2

## Abstract

This study investigated the impact of criterion-based vs. social reference frames on behavioural and neural correlates of performance monitoring while taking individual differences in control beliefs into account. We conducted two experiments administering a time estimation task in which feedback was either delivered pertaining to participants’ own performance (nonsocial/criterion-based reference) or to the performance of a reference group of previous participants (social reference). In Experiment 1, 34 male volunteers participated. To test generalizability of the observed results to both sexes/genders, we recruited 36 female volunteers for Experiment 2. P2 and P300 amplitudes were generally larger in social than in nonsocial reference trials in the male participants of Experiment 1. ΔFRN amplitudes were larger for social compared to non-social reference trials in Experiment 1. No effects of reference frame were found in the female sample of Experiment 2. Rather, P2 and ΔFRN effects showed opposing patterns for nonsocial versus social reference frames. However, stronger internal control beliefs were accompanied by larger FRN amplitudes of negative social reference trials in both samples, suggesting generalizable effects independent of sex/gender. Enhanced P2 and ΔFRN amplitudes for social versus nonsocial reference trials suggest enhanced attentional capture and higher saliency of socially framed feedback in male participants only. In both sexes/genders, however, the social reference frame possibly challenges internal control beliefs and by this enhances performance monitoring. Our results demonstrate the complex interplay of trait variables and reference frames during performance monitoring influencing our daily lives-reference frames are omnipresent in education and one’s working environment.

## Introduction

Monitoring other people’s behaviour entails comparing our behaviours to those of our fellow humans, such as when we are required to adapt or coordinate them to learn a new skill or to achieve a common goal. In this way, our fellows form a social comparison group and a social reference frame that constitutes the basis for obtaining performance feedback (applying a relative feedback standard). In contrast, there are behaviours in which the reference frame for performance feedback is set by a criterion defined irrespective of any social context (i.e., a nonsocial reference frame, applying an absolute feedback standard). Imagine, for example, writing a math exam in school with a maximum of 100 points. Using a social reference frame, the teacher could calculate a distribution of students’ performances after the exam. The point range for each grade and thus individual performance will be evaluated with respect to performance of the whole class—hence explicitly inducing social comparison processes. In contrast, referring to a nonsocial (criterion) reference frame, the teacher could announce the point range for each grade before the exam and each student will be graded based on this predefined criterion. Accordingly, depending on the frame of reference applied and the reference group, identical individual performances could result in different grades and thus performance feedback. This leads to the question how social versus nonsocial criterion reference frames exert influence on task performance. Importantly, individuals’ sensitivity to different reference frames might be influenced by stable dispositional traits, such as attribution styles—how people tend to explain causes of events and behaviours (Rotter, 1966). Thus, the current study addressed this research question by assessing behavioural and neuronal correlates, as well as personality traits to investigate possible effects and underlying mechanisms of state and trait variables during performance monitoring in a social context.

We used a modified time estimation task (Miltner, Braun, & Coles, 1997) to provide participants with performance feedback, which was given either in relation to a social or a nonsocial criterion reference frame. Participants’ task was to estimate the duration of 1 second by delivering a button press whenever they thought that 1 second had elapsed. During the nonsocial reference frame condition, participants were informed that feedback was constantly adjusted based on their own performance in relation to a specific criterion; the estimation time windows were narrowed down or enlarged based on their previous estimations. During the social reference frame condition, participants were informed that feedback was constantly adjusted based on their performance in relation to a social reference group. Comparing their estimations to mean estimation times of a group of previous participants while the estimation time windows also were constantly adjusted in relation to the reference group. Thus, both conditions were applying an adaptive set-up for feedback delivery, only differing in the respective reference frame.

In addition to reaction time data reflecting time estimation, we measured event-related potentials (ERPs) to assess cognitive processes associated with feedback processing with high temporal resolution. Approximately 200–300 ms after feedback onset, the Feedback-Related Negativity (FRN; Miltner, et al., 1997) was assessed at frontocentral electrode sites. The FRN is a negative deflection being more pronounced after negative/incorrect compared with positive/correct (Miltner, et al., 1997; Nieuwenhuis, Yeung, Holroyd, Schurger, & Cohen, 2004) or unexpected compared with expected feedback outcomes (Alexander & Brown, 2011; Hajcak, Moser, Holroyd, & Simons, 2007; Pfabigan, Alexopoulos, Bauer, & Sailer, 2011). The anterior midcingulate cortex (aMCC) and the ventral striatum are discussed as potential neuronal generators of the FRN (Becker, Nitsch, Miltner, & Straube, 2014; Debener et al., 2005). It is assumed to reflect an unsigned prediction error signal (Hayden, Heilbronner, Pearson, & Platt, 2011; Talmi, Atkinson, & El-Deredy, 2013) important during learning processes. Before the FRN component, a positive deflection at frontal electrode sites peaking around 180 ms after feedback onset has gained additional interest in ERP research on feedback processing and performance monitoring. The P2 component is assumed to reflect early stages of attention capture and processing of the affective significance of target stimuli (Cuthbert, Schupp, Bradley, Birbaumer, & Lang, 2000; Potts, 2004; Potts, Martin, Burton, & Montague, 2006). Since FRN and P2 components overlap and influence each other’s amplitude variation, it is important to demonstrate experimental effects in both of them. Approximately 300–500 ms after feedback onset, another positive deflection, the P300 component (Duncan Johnson & Donchin, 1977; Polich, 2007) occurs with a maximum usually located at medial parietal electrode sites. In the feedback context, P300 amplitudes are assumed to index more elaborate stimulus processing, related to processes such as motivational saliency and context updating in working memory (Bellebaum & Daum, 2008; Nieuwenhuis, Aston-Jones, & Cohen, 2005).

Current research offers several mechanisms driving possible differences between social and nonsocial reference frames. One refers to perceived causality of action, i.e., whether participants experience feedback as directly caused by their own actions or not. Humans have the tendency to think about and assign causes to all possible situations. This behavioural tendency is known as causal attributions (Heider, 1958) and often constitutes of automatic inferences about causes of events in social situations, facilitating our understanding of social interactions (Weiner, 1985). Internal attributions are elicited during situations in which we feel responsible for an event, while external attributions are elicited when we assign the cause for an event to other individuals or contextual circumstances. (Rotter, 1966). A nonsocial reference frame should imply high, whereas a social reference frame should imply lower perceived causality since participants’ feedback also is dependent on other individuals’ actions. Thus, one could assume that the individual together with the social reference group share responsibility for her/his respective feedback outcome. The sharing of responsibility leads to the social phenomenon of diffusion of responsibility, which describes the observation that feelings of personal responsibility and accountability become diminished in case individuals work together as compared to working alone (Darley & Latane, 1968; Forsyth et al., 2002). Studies on shared responsibility have shown diminished feedback ERPs when sharing responsibility compared with being the only one responsible for feedback outcomes (Beyer et al., 2016; Hewig et al., 2008; Li et al., 2010). These diminished ERP amplitudes have been interpreted as correlates of decreased responsibility for the outcomes due to less controllability and accountability (Coricelli et al., 2005; Walton et al., 2004). Of note, these studies did not consider individual differences and dispositions in personality factors related to control and accountability. In this respect, individual attribution styles seem particularly relevant, because they might interact with the perceived causality of an action. Attribution style refers to an individual disposition to apply rather more internal or more external attribution strategies (Rotter, 1966). So far, only one study has reported an association between feedback-related ERPs and attribution style (Aarts & Pourtois, 2012). The authors observed more pronounced FRN amplitudes in participants who attributed their behaviour internally, suggesting that participants with higher generalized internal locus of control scores (Rotter, 1966) may more easily integrate external evaluation, such as feedback stimuli with their internally generated actions. For the current experiment, we thus predicted that feedback during the nonsocial reference condition would be easier to integrate than feedback during the social reference condition.

Additional aspects of potential relevance for socially framed performance feedback are social comparison and evaluation processes. Although participants were explicitly instructed that the task setting was not intended to induce a competitive set-up, it is plausible to assume that social comparison and evaluation processes have implicitly arisen. Wu et al. (2012) have shown that only P300, but not FRN, amplitudes were indicative of social comparison in a dot-estimation task while participants were comparing performance feedback of their own dot-estimation to that of an anonymous player. In contrast, Luo et al. (2015) also reported FRN amplitude variation in response to social comparison in a lottery gambling task with two anonymous players in which monetary outcomes of all three players were displayed concurrently. FRN amplitudes were larger when participants received different feedback than the other two players. Other studies directly induced social evaluation via applying tasks in which participants had to judge whether unknown individuals had given spontaneous “like” or “dislike” judgements when presented with their faces (Somerville et al., 2006), with mixed findings. No FRN effects were observed by van der Molen et al. (2013). Others reported expectancy violation effects when participants’ judgements did not match those of the unknown individuals (Dekkers et al., 2015; van der Molen et al., 2016; van der Veen et al., 2016). Also, larger FRN amplitudes for unfavourable compared with favourable judgements were found (Kujawa et al., 2014; Sun & Yu, 2014). Based on these studies, we speculated that if implicit comparison processes would be triggered by the social reference frame condition, they should yield enhanced ERP amplitudes in the social reference condition.

The current experiment allowed to test and disentangle two hypotheses with opposing predictions. If perceived causality of action is relevant in the current manipulation, one would expect enhanced P2, FRN, and P300 amplitudes for the nonsocial compared with the social reference frame condition, due to less controllability/accountability during the social reference condition (Beyer et al., 2016; Li et al., 2010). Moreover, participants with higher generalized internal locus of control scores (higher internal control beliefs) should show enhanced FRN amplitudes in the nonsocial reference condition, in which their actions were directly connected to the subsequent feedback (Aarts & Pourtois, 2012). In contrast, another hypothesis would suggest that the social reference frame will be perceived *per se* as more salient than the nonsocial one because of the high prevalence and specific relevance of implicit social comparison and evaluation processes (Dunbar, 2011). This hypothesis would thus predict enhanced P2, FRN, and P300 amplitudes during the social compared with the nonsocial reference frame condition (Luo et al., 2015; Sun & Yu, 2014; Talmi et al., 2013). Individual differences in attribution style also could be relevant in this regard, but it was less clearly predictable how they would modulate performance monitoring.

We conducted two experiments to address our research question. Experiment 1 tested solely male participants. However, current research suggests at least small effects of sex/gender on performance monitoring (Fischer et al., 2016; Larson et al., 2011), and a recent social judgement study also reported sex/gender differences (van der Veen et al., 2016). This study further suggested considering sex/gender as an important factor contributing to individual differences in the domain of social feedback research. We thus conducted a follow-up experiment to test whether results from Experiment 1 were generalizable to a female sample.

## Experiment 1

### Methods

#### Participants

Thirty-four male volunteers (mean age 26.2 years, standard deviation [SD] = 5.23, range 20-40) took part in this electroencephalography (EEG) study. All participants were right-handed as assessed via a handedness inventory (Oldfield, 1971), had normal or corrected-to-normal vision, and reported no past or current psychiatric or neurological disorder. Sample size was based on a priori power considerations and analysis showing that a sample of at least 30 participants would have sufficient power (1-β > 0.80) to detect medium to large effects (Luo et al., 2015) of the important comparisons (*η*_*p*_^*2*^ = 0.12, as in SPSS; G*Power 3.1; Faul et al., 2009). Thus, we recruited slightly more participants to be prepared for potential drop outs. Written, informed consent was obtained prior to the experiment. The study conformed to the Declaration of Helsinki (7^th^ revision, 2013) and was approved by the ethics committee of the University of Vienna. Participants performed an additional experimental task before the current one, which was not related to the present one and will be reported elsewhere.

#### Questionnaires

Before EEG data collection, participants filled in online questionnaires including the Brief Symptom Inventory (BSI; Derogatis & Melisaratos, 1983) to exclude participants with heightened psychiatric symptoms (cut-off sex-specific: only participants with T-values < 60 were included). The adapted German version of Levenson’s IPC scale (Levenson, 1981) was administered after task completion (FKK - Fragebogen zu Kompetenz- und Kontrollüberzeugungen; Krampen, 1991). This questionnaire consists of 32 items asking for individual opinions on 6-point Likert scales from strongly disagree (−3) to strongly agree (+3). In each case, eight items form the following subscales: self-concept of own competence (FKK-SK), generalized internal control beliefs (FKK-I), powerful others’ control beliefs (FKK-P), and chance control beliefs (FKK-C). Internal reliability of the subscales is acceptable to good (0.70-0.89). Particularly relevant for the current study was the subscale assessing generalized internal control beliefs (FKK-I)—describing generalized control over one’s own life and events in the person-specific environment.

#### Task

**Figure 1.**
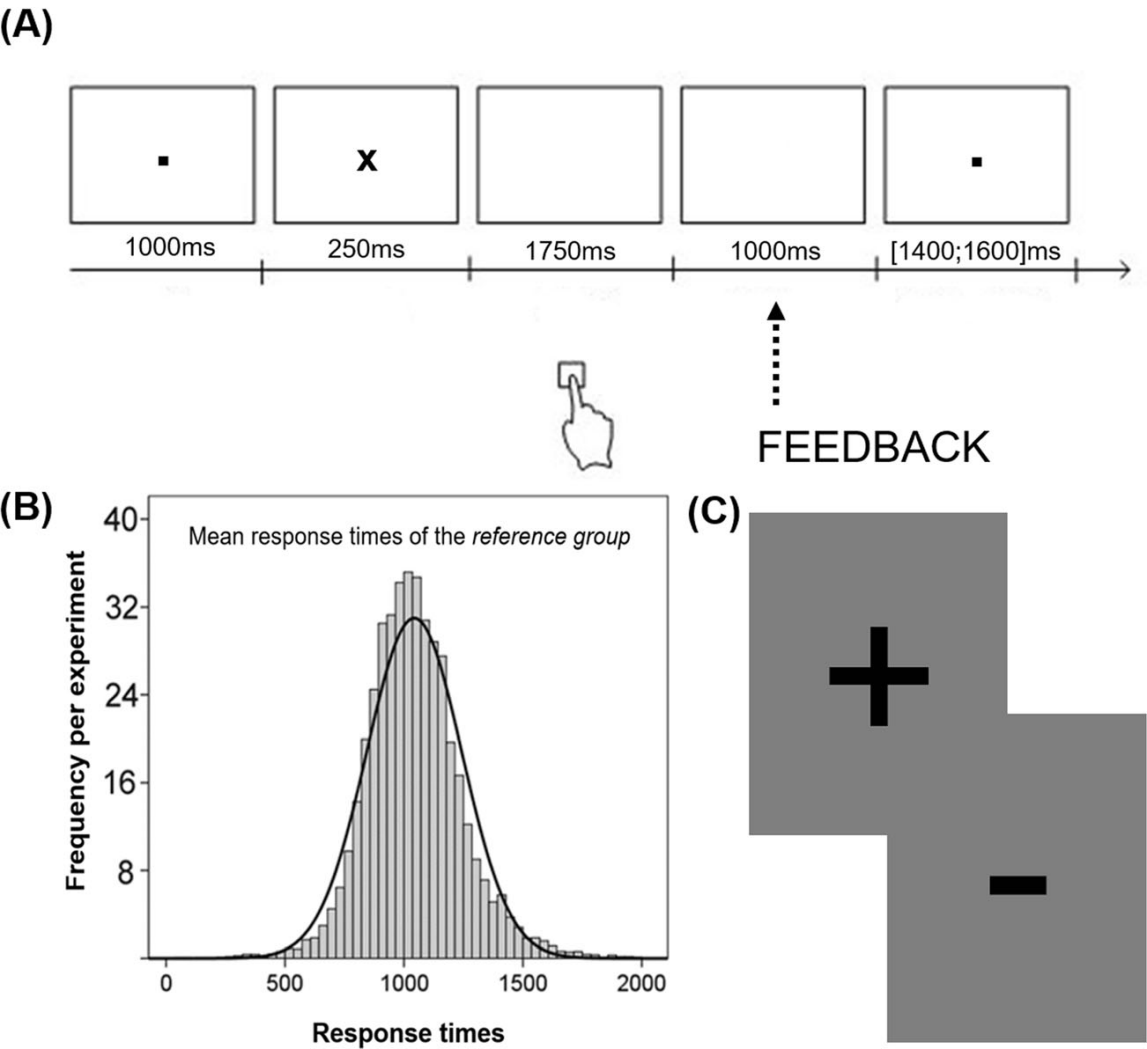
**A** The time line of the time estimation task. Participants indicated their estimations via button press in the 1,750-ms time period after cue (“x”) offset. **B** The histogram of average response times of the group of previous participants used for demonstration purposes during instruction. **C** Feedback stimuli used to indicate correct (plus) and incorrect (minus) time estimations.

In a modified version of a time estimation task (Miltner et al., 1997; Pfabigan et al., 2014, 2015a, 2017), participants were required to estimate the passing of 1 second and to indicate their time estimation via button press. Both experimental conditions (social vs. nonsocial reference) had in common that each trial started with the presentation of a central black fixation dot. After 1,000 ms, a black star replaced the dot for 250 ms and indicated the starting point of each time estimation. Subsequently, a blank grey screen was presented for 1,750 ms, during which participants indicated the estimated elapse of 1 second via button press (button 1 on a standard keyboard; right index finger); 2,000 ms after the onset of the star, feedback was presented for 1,000 ms to demonstrate time estimation accuracy. Feedback stimuli consisted of black plus and minus symbols (positive/negative feedback). Feedback stimuli were equiluminiscent and comparable in size (4 x 5 cm) and were used in a previous study (Pfabigan et al., 2014). The subsequent intertrial-interval depicted the black fixation dot (duration 1,400-1,600 ms; Figure 1). Feedback was provided based on individual performance. However, task difficulty (the time window for correct estimations) was adjusted to the individual performance level to guarantee approximately comparable numbers of correct and incorrect trials per condition. Importantly, for both reference conditions, participants were explicitly informed about the adaptive nature of the task and the exact rules. For the nonsocial reference condition, participants’ current estimations were compared with their estimations in preceding trials. Each participant started with the following criteria: positive feedback was given in cases in which the button press fell in the time window of 900 to 1,100 ms after the onset of the star. Subsequently, the width of this time window was automatically adjusted based on individual performance on the preceding trial (Miltner, et al., 1997). After a trial with positive feedback (i.e., a correct time estimation), the time window was narrowed down by 10 ms at both ends of the window (e.g., 910-1,090 ms after the initial trial). Thus, to receive positive feedback in the next trial, the estimation had to be closer to the 1,000-ms goal than the previous one. After a trial with negative feedback (i.e., an incorrect time estimation), the time window became wider again by adding 10 ms at both ends, thus making it easier to receive positive feedback in the next trial. Consequently, the overall probability of positive and negative feedback was approximately 50% for all participants. An adaptive set-up also was used for the social reference condition. Specifically, participants were told that their time estimations were compared to the average estimations of a group of previous participants in our laboratory. As depicted in Figure 1B, participants were presented with a figure showing time estimation behaviour (mean response times and their Gaussian normal distribution) of 162 previous participants, whose data were collected within the past 5 years in different projects. In addition, the current participants were told that their time estimation was compared to the medium 50% of the reference group in the beginning. After a trial with positive feedback (i.e., a correct time estimation), the size of the medium reference group for comparison would be narrowed down by 2% (48%). Thus, to receive positive feedback in the next trial, the estimation had to be closer to the 1,000-ms goal than the previous one. After a trial with negative feedback (i.e., an incorrect time estimation), the size of the medium reference group for comparison would be enlarged by 2% again, thus making it easier to receive positive feedback in the next trial. This description was chosen to imply that the criteria used in the social reference condition match those of the nonsocial reference condition in terms of ±10-ms changes and initial window size of 900–1,100 ms. Thereby, performing the task more similarly to the reference group was equivalent to more accurate time estimations towards the 1,000-ms goal. Again, overall probability of positive and negative feedback was approximately 50% for all participants.

Importantly, participants were informed that both conditions were comparable concerning task difficulty and that no competitive situation was intended to be introduced by the reference group. Of note, we used the same adaptive algorithm based on individual performance (Miltner et al., 1997) in both conditions. Nevertheless, in the social reference frame condition, participants were led to believe that their time estimations were compared to the reference group estimations. Thereby, we introduced a critical aspect of social reference groups, for which one’s own success is highly dependent on the standards of the reference frames/groups (and less dependent on objective performance per se). The respective 100 trials, including social reference frame or nonsocial reference frame feedback, were presented block-wise as previously suggested (Pfabigan et al., 2014) with a short break in-between. Half of the participants started with the social reference frame condition, the other half with the nonsocial reference frame condition. The experiment consisted of 10 training and 200 experimental trials (100 per condition). Overall, EEG data collection lasted approximately 20 minutes. As manipulation checks, participants were asked to provide ratings on 7-point Likert scales concerning subjectively experienced attention, guilt, contribution, controllability, distress, effort, feelings after positive and negative feedback, interest, and satisfaction after the experiment—asked separately for both reference frame conditions (presentation randomized). At the end of the experiment, they received a financial remuneration of €25 for their participation.

#### Electrophysiological recording and data analysis

Stimulus presentation was controlled by E-Prime 2.0 (Psychology Software Tools, Inc., Sharpsburg, PA). Participants were seated in a shielded chamber approximately 70 cm in front of a 19” CRT monitor (Sony GDM-F520; 85 Hz refresh rate). EEG was recorded via 59 Ag/AgCl ring electrodes mounted in a fabric cap (EASYCAP GmbH, Herrsching, Germany; model M10). Additionally, four electrodes were placed 1 cm above and below the left eye and on the outer canthi to measure horizontal and vertical electrooculogram (EOG). EEG signals were collected with a DC amplifier set-up (NeuroPrax, neuroConn GmbH, Ilmenau, Germany), referenced online against an additional electrode on the forehead serving as ground electrode, and sampled at 500 Hz for digital storage.

EEG data were analysed using EEGLAB 13_1_1b (Delorme & Makeig, 2004) implemented in Matlab 2014a. Offline, high pass (0.1 Hz) and low-pass filters (cut-off frequency 30 Hz, roll-off 6dB/octave) were applied to the EEG data. Data were re-referenced to linked mastoids and extended infomax independent component analysis (ICA; Bell & Sejnowski, 1995) was applied to detect eye movement-related artefacts. After discarding these artefacts (on average two components per participant; range 1-4), data segments of the four possible conditions were extracted starting 200 ms before feedback onset and lasting for 1,200 ms (*social positive*, *social negative*, *nonsocial positive*, and *nonsocial negative*). Subsequently, semi-automatic artefact correction was conducted in EEGLAB. Trials with voltage values exceeding ±75 μV (function *pop_eegthres*) or voltage drifts > 50 μV (function *pop_eegrejtrend*) were automatically marked by the algorithms. These trials were rejected in case visual inspection also indicated artefact affliction. Artefact-free segments were averaged participant- and condition-wise; on average 41.44 (*SD* = 8.00) trials per condition. To keep the statistical model simple and to increase ERP signal-to-noise ratio (Luck & Gaspelin, 2017), we assessed P2, FRN, and P300 peaks at clusters of several merged electrodes applying a region of interest approach. An electrode cluster including FCz and six surrounding electrodes (R11, R14 [FCz], R15, L8 [Fz], L9, L12, L16 [Cz]) was used for assessing P2 and FRN components (Gu et al., 2011; Pfabigan et al., 2015b), a cluster including Pz and CPz and two electrodes in between (R24 [CPz], R25, L22, L26 [Pz]) for the P300 component.

The most positive peak in the time window 130-230 ms after feedback onset was selected and its value extracted as P2 peak. For the FRN component, the most negative peak in the time window 230-400 ms after feedback onset was selected. To quantify FRN amplitude variation, we subtracted this FRN peak from the preceding P2 peak to assess the FRN component as a difference value (i.e., as a peak-to-peak measure) to account for potential component overlap (Holroyd et al. 2003; Pfabigan et al., 2011). For the P300 component, the most positive value in the time window 250-600 ms after feedback onset was selected and extracted. A winsorizing procedure (Wilcox 2012) was applied per condition for ERP data and changes in time estimation response times to account for outliers. As discussed by Keselman et al., (2003) and Wilcox (2010), including outlier values in the analysis violates assumptions of general linear model estimations and should be avoided. All values larger than the value corresponding to the 75th percentile plus 1.5 times the interquartile range per condition were replaced with the maximum amplitude within this range in the corresponding condition. Accordingly, mean values smaller than 25th percentiles minus 1.5 times the interquartile range per condition were replaced with the minimum amplitude within this range in the correspondent condition.

Statistical analyses were performed using PASW 18 (SPSS Inc., IBM Corporation, NY) and Statistica 6.0 (StatSoft Inc., Tulsa, OK). Significant interaction effects were explored with HSD Tukey post-hoc tests and planned comparisons, whenever *a priori* hypotheses existed. Associations between FKK-I scores and ERPs were calculated with Spearman correlations per component. Potential differences between correlation coefficients were tested using Steiger’s Z test (Steiger, 1980). The significance level was set at *p* < 0.05. Partial eta-squared (*η*_*p*_^*2*^) is reported to indicate effect sizes for significant ANOVA results. Values of *η*_*p*_^*2*^ = 0.01, *η*_*p*_^*2*^ = 0.06, and *η*_*p*_^*2*^ = 0.14 represent small, medium, and large effects (Kirk, 1996). For t-tests, Cohen’s d is reported (Cohen, 1988).

#### Behavioural data analysis

Differences in response times were calculated per participant and condition between each trial and its preceding trial to describe changes in response times evoked by directly preceding feedback. These mean trial-to-trial changes were further separated for trials in which reaction time changes were classified as correct adjustments (i.e., the current estimation was closer to 1,000 ms than the previous one representing a successful response) and those classified as incorrect adjustments (i.e., the current estimation was farther away from 1,000 ms than the previous one, representing an unsuccessful response). Postexperimental ratings were analysed separately using dependent t-tests with the factors *reference frame.*

### Results

#### Behavioural results

Tables 1 and 2 summarize mean and standard deviations of behavioural, rating, and questionnaire data. Corroborating task validity, trial-to-trial changes in response times were affected by *feedback valence* (F(1,33) = 316.23, *p* < 0.001, *η*_*p*_^*2*^ = 0.91) and *estimation adjustment* (F(1,33) = 65.61, *p* < 0.001, *η*_*p*_^*2*^ = 0.67), and their interaction (F(1,33) = 126.47, *p* < 0.001, *η*_*p*_^*2*^ = 0.79). No other effects reached significance (all *p* > 0.233). A Tukey post-hoc test for the interaction showed that trial-to-trial changes in response times were smallest after correct adjustments following positive feedback (all *p* < 0.001) and largest after correct adjustments following negative feedback (all *p* < 0.027). Moreover, following negative feedback, trial-to-trial changes were larger for correct than incorrect adjustments (*p* = 0.027); while following positive feedback, trial-to-trial changes were larger for incorrect than correct adjustments (*p* < 0.001).

**Table 1.** Sample characteristics

		Experiment 1		Experiment 2		Comparison Experiments 1/2	
		*M*	*SD*	*M*	*SD*	*t*	*p*
Age		26.18	5.23	25.58	5.17	0.48	0.64
BSI	*Raw scores*	9.29	5.66	13.14	7.25		
	*T values*	45.94	7.42	46.06	7.87	-0.62	0.95
FKK							
	*SK*	34.91	5.46	33.53	7.06	0.92	0.36
	*I*	35.03	4.03	33.08	4.31	1.95	0.06
	*P*	23.97	5.79	23.81	5.15	0.13	0.90
	*C*	21.82	4.91	22.17	4.86	-0.29	0.77

**Table 2.** Behavioral and rating data

		Experiment 1				Experiment 2			
		*M*	*SD*			*M*	*SD*		
Estimation time window (ms)	Social pos	139.21	50.87			134.74	42.28		
	Social neg	127.58	46.34			127.15	45.36		
	Non-social pos	131.98	32.82			137.96	41.57		
	Non-social neg	123.85	33.19			128.40	41.63		
Ratio over-/underestimations	Social	1.11	0.41			1.23	0.85		
	Non-social	1.15	0.39			1.17	0.44		
		Successful		Unsuccessful		Successful		Unsuccessful	
		*M*	*SD*	*M*	*SD*	*M*	*SD*	*M*	*SD*
Deviation 1,000-ms goal (ms)	Social pos	41.47	13.45	185.72	51.14	41.54	13.74	180.40	54.60
	Social neg	117.72	40.50	329.06	101.52	120.72	51.52	330.47	100.67
	Non-social pos	45.70	18.22	187.97	62.75	42.96	15.55	190.60	65.73
	Non-social neg	123.82	56.94	350.96	129.14	124.35	52.93	339.43	136.55
Deviation 1,000-ms goal (SD)	Social pos	32.02	10.93	123.71	45.25	32.35	9.78	119.44	50.92
	Social neg	90.72	33.59	143.42	51.69	89.97	39.33	148.90	51.08
	Non-social pos	32.32	12.97	124.23	44.94	30.37	9.19	125.72	45.16
	Non-social neg	97.31	46.70	152.12	58.45	98.12	44.41	151.72	59.86
Trial-to-trial changes (ms)	Social pos	69.27	22.10	162.63	56.07	76.25	24.11	156.68	43.27
	Social neg	231.41	60.63	208.71	69.27	225.50	64.08	223.53	83.92
	Non-social pos	76.15	21.50	159.65	45.70	74.55	19.91	165.17	54.10
	Non-social neg	222.91	54.72	205.02	64.36	233.44	83.41	214.22	85.23
Postexperimental ratings		Social context		Non-social context		Social context		Non-social context	
		*M*	*SD*	*M*	*SD*	*M*	*SD*	*M*	*SD*
	Attention	6.97	2.08	7.62	1.71	6.69	1.69	6.97	1.83
	Guilt	5.94	2.53	6.53	2.35	5.36	2.63	5.67	2.78
	Contribution	6.41	1.83	6.91	1.96	6.08	1.40	6.92	1.42
	Controllability	4.82	2.12	5.35	2.45	4.42	2.02	4.81	1.88
	Distress	5.06	2.68	4.88	2.53	4.64	2.33	5.53	2.14
	Effort	8.09	1.68	8.15	1.37	8.17	1.50	8.08	1.63
	Feelings positive feedback	7.12	1.39	7.03	1.19	7.11	1.37	7.19	1.43
	Feelings negative feedback	4.18	1.64	3.74	1.16	3.92	1.20	3.36	1.05
	Interest	6.76	1.83	6.59	2.09	5.92	2.13	6.19	2.32
	Satisfaction	5.68	2.31	5.41	2.26	5.11	1.91	5.28	1.94

Compared with the social reference condition, the nonsocial one yielded higher attention ratings (t(33) = −2.24, *p* = 0.032, d = 0.39). The other ratings did not differ significantly for the two reference frames (all *p* > 0.117).

#### EEG results

The FRN ANOVA showed significant main effects of *feedback valence* (F(1,33) = 59.54, *p* < 0.001, *η*_*p*_^*2*^ = 0.64), with larger FRN amplitudes for negative than positive feedback, and *reference frame* (F(1,33) = 5.85, *p* = 0.021, *η*_*p*_^*2*^ = 0.15), with larger FRN amplitudes for social than non-social feedback. The interaction *reference frame x feedback valence* (F(1,33) = 6.39, *p* = 0.016, *η*_*p*_^*2*^ = 0.16) was significant. The FRN amplitude difference between negative and positive feedback (ΔFRN) was significantly larger for social than nonsocial reference trials (t(33) = 2.53, *p* = 0.016, d = 0.45). A significant positive correlation was observed for social negative trials and FKK-I scores (r_s_ = 0.394, *p* = 0.021), whereas nonsocial negative trials were not associated with generalized internal control beliefs (r_s_ = 0.123, *p* = 0.488). Steiger’s Z test showed that these two correlation coefficients differed significantly from each other (z = 2.32, *p* = 0.020), thereby suggesting that higher FKK-I scores were solely associated with larger FRN amplitudes for negative social reference feedback. Positive feedback was not associated with FKK-I scores (both *p* > 0.488).

The P2 ANOVA showed significant main effects of *feedback valence* (F(1,33) = 29.51, *p* < 0.001, *η*_*p*_^*2*^ = 0.42), with larger P2 amplitudes for positive than negative feedback, and *reference frame* (F(1,33) = 8.51, *p* = 0.006, *η*_*p*_^*2*^ = 0.21), with larger P2 amplitudes for social than non-social feedback. Their interaction was not significant (F(1,33) = 0.40, *p* = 0.531). P2 amplitudes were not significantly correlated with FKK-I scores (all *p* > 0.185).

The P300 ANOVA also showed significant main effects of *feedback valence* (F(1,33) = 27.94, *p* < 0.001, *η*_*p*_^*2*^ = 0.46), with larger P300 amplitudes for positive than negative feedback, and of *reference frame* (F(1,33) = 4.93, *p* = 0.033, *η*_*p*_^*2*^ = 0.13), with larger P300 amplitudes for social than non-social feedback. Their interaction was not significant (F(1,33) = 0.05, *p* = 0.829). P300 amplitudes were not significantly correlated with FKK-I scores (all *p* > 0.352).

**Figure 2.**
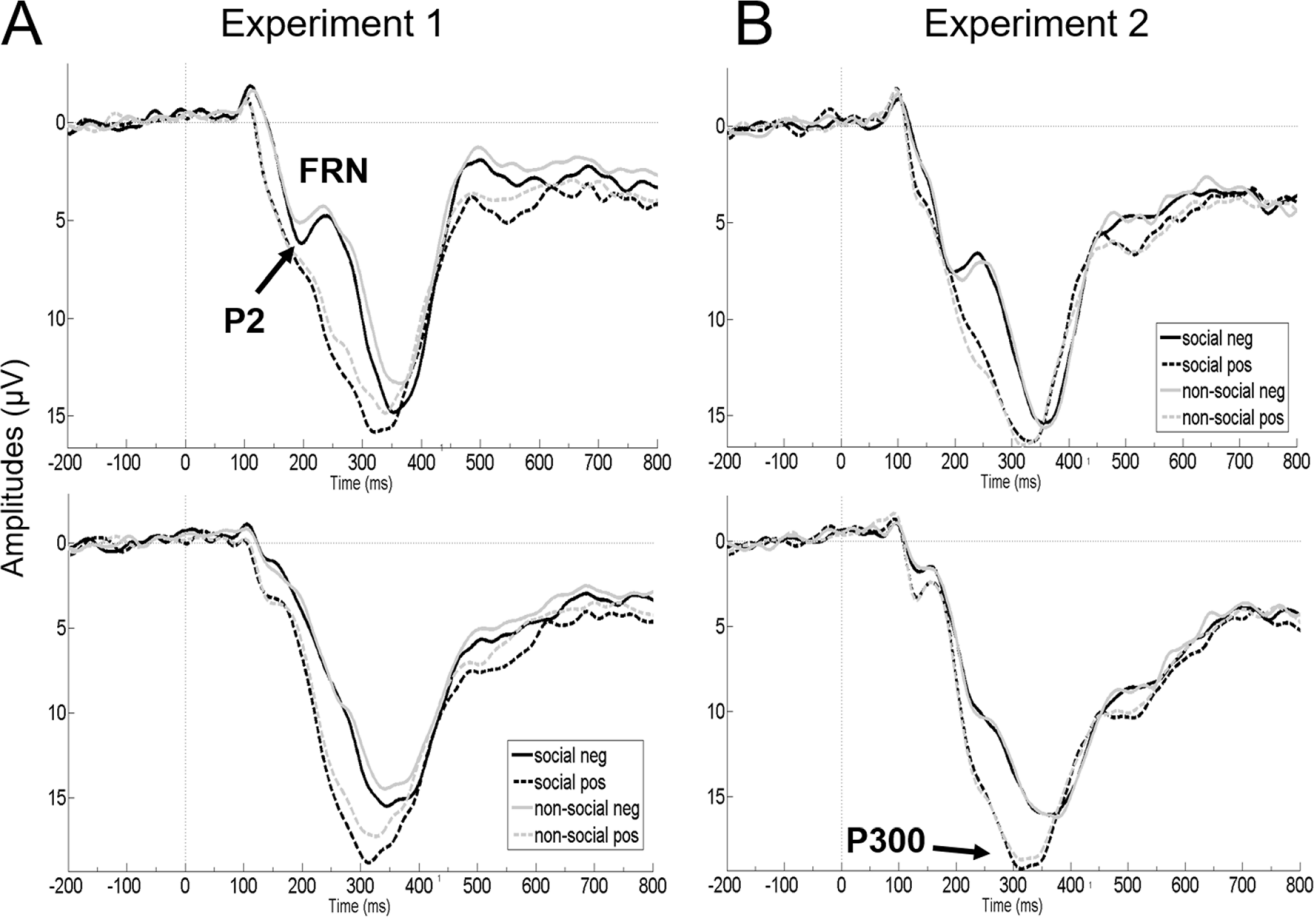
Amplitude courses of P2 and FRN components at the frontal electrode cluster (upper panel) and of the P300 component at the parietal electrode cluster (lower panel), separately plotted for Experiment 1 (A) and Experiment 2 (B). Feedback onset at 0 ms; negative is drawn upward per convention.

See Table 3 for descriptive statistics and Figure 2A.

**Table 3.** Electrophysiological data in μV

	Experiment 1					
	FRN		P2		P300	
	*M*	*SD*	*M*	*SD*	*M*	*SD*
Social pos	2.31	1.92	11.95	5.60	20.03	5.33
Social neg	6.49	3.72	9.38	4.53	17.36	5.74
Non-social pos	2.35	1.54	10.77	5.08	18.86	4.72
Non-social neg	5.13	3.42	7.72	4.91	16.33	5.91
	Experiment 2					
	FRN		P2		P300	
	*M*	*SD*	*M*	*SD*	*M*	*SD*
Social pos	2.41	1.97	11.29	5.72	20.73	4.92
Social neg	5.01	2.52	9.35	4.23	18.40	6.41
Non-social pos	2.42	1.78	12.61	5.01	20.59	4.40
Non-social neg	5.64	3.20	9.89	4.38	18.70	5.65

## Discussion Experiment 1

On the neural level, all feedback ERPs were sensitive to the reference frame manipulation. P2 and P300 amplitudes were generally enhanced during social compared with nonsocial reference feedback. FRN amplitude enhancement for social compared to nonsocial feedback was observed for the difference between negative and positive feedback. Moreover, generalized internal control beliefs were positively associated with FRN amplitudes in negative social compared with negative nonsocial reference feedback. Postexperimental questions tapping into self-reported affect showed only small differences in how participants dealt with feedback during the two reference frames, and no differences in behavioural performance were observed

Addressing our two opposing hypotheses, neural correlates failed to support the assumption of a sort of diffusion of responsibility effect of the current manipulation. This result might be because the current reference group manipulation did not introduce a simultaneous (online) interaction with other individuals since data of the social reference group were collected before the current experiment. Previous EEG studies investigating diffusion of responsibility directly introduced co-players to their participants who interacted with each other simultaneously (Beyer et al., 2016; Hewig et al., 2008; Li et al., 2010). The current experimental set-up did not allow an “online” interaction with members of the reference group, which was further described rather broadly and in an abstract way. Diffusion of responsibility effects might only be induced in case one believes to directly interact with other individuals compared with offline interaction. Future studies should test whether the current reference manipulation would yield diffusion of responsibility-like effects when the social reference frame is defined by a second participant, who is physically present during the experiment.

Although only virtually established, the social reference group manipulation nevertheless influenced several stages of feedback processing, which corroborates notions of higher feedback salience during the social than the non-social frame condition in our participants. Implicit social evaluation and comparison processes could have been introduced by the current setting despite specific instructions to neglect them. Our manipulation did not allow for upward or downward comparison with the social reference group as previous studies did (Lindner et al., 2015). However, when comparing one’s own performance to group performance, negative social feedback could have reflected a kind of deviance from the social group, whereas positive social feedback could have reflected an affiliative signal of the group. Indeed, several studies addressing social conformity—a behavioural tendency to change one’s own behaviour in line with group behaviour (Cialdini & Goldstein, 2004)—reported enhanced FRN amplitudes in response to a mismatch between individual and group opinions (Kim et al., 2012; Shestakova et al., 2013). The authors interpreted their findings in light of reinforcement learning theories (Holroyd & Coles, 2002), suggesting FRN amplitude variation to reflect reward prediction error signals also in the social domain. This fits with our data, because reference frame effects were observed for ΔFRN (i.e., the difference between negative and positive feedback trials), which often is calculated to represent reward prediction error signals (Holroyd & Coles, 2002). Participants might have experienced the discrepancy between negative and positive social reference feedback inherently as more salient than the one for nonsocial reference feedback because they might have expected to perform “on the same level” as the social reference group. Along these lines, enhanced FRN amplitudes were also observed for social evaluation situations without additional expectancy manipulations (Kujawa et al., 2014; Sun & Yu, 2014).

Already initial attention capture by the feedback stimuli was influenced by the reference frame as well as feedback valence. P2 enhancement indicated that in general social reference frame and positive feedback stimuli led to enhanced attention allocation (Potts, 2004). The observation of larger P300 amplitudes for social compared to nonsocial reference frame trials corroborates social evaluation and judgement studies (Dekkers et al., 2015; Wu et al., 2012) suggesting heightened motivational significance of social reference frame trials also for more elaborate cognitive processing. In line with these studies, we hypothesize that the social reference frame was more salient than the nonsocial one. It might have implicitly triggered social comparison processes, which tagged delivered feedback with additional motivational saliency. Interestingly, subjective ratings after the experiment showed higher reports of attention for the non-social than the social reference condition, thereby suggesting that subjective experience was prone to a post-hoc interpretation bias when assessed offline after task completion.

Although the current reference frame manipulation affected correlates of early attentional (P2), coarse evaluation/categorization (FRN), as well as more elaborate cognitive processes (P300), no behavioural effects were observed. In a previous study, we argued that the time estimation task is a rather simple cognitive task during which individual performance is immune against external manipulations (for example, the manipulation of physical stimulus aspects; Pfabigan et al., 2015a, 2017). Social context manipulations exerting top down influence on task correlates also might be too weak to influence individual behaviour during this task.

Regarding individual dispositions of attribution styles, we observed a positive association of generalized internal control beliefs and FRN amplitudes exclusively for the social negative reference condition. Enhanced scores of controllability of one’s own life were associated with more pronounced feedback saliency signals during the social comparison context for negative feedback. This is opposed to our assumption that internal control beliefs would be related to FRN variation in the non-social reference condition in which behaviour could be directly attributed to internal causes (Aarts & Pourtois, 2012). This might be explained by the current manipulation applying a within-subject manipulation in which participants knew beforehand that they would encounter social and non-social reference trials. Individuals who strongly believe in their ability to control their own lives and their environment might experience a social comparison context as challenge or even threat to their internal control believes because the relative feedback standard induced performance uncertainty. Since the exact performance of the social reference group was unknown it might have been more difficult to assess the quality of one’s own performance. Therefore, participants with more pronounced internal control beliefs might have exhibited enhanced FRN amplitudes, i.e., enhanced performance monitoring, for negative feedback in the social reference condition to compensate for this uncertainty. Indeed, social comparisons in general might be perceived as more ambiguous than non-social ones because they often fail to contain specific information regarding the performance level of the comparison group. For example, the current manipulation explained the adaptive nature and the related changes of the size of the reference group but did not introduce exact numbers as in the nonsocial context. This information was only introduced implicitly by telling that both experimental conditions were comparable regarding task difficulty. Thus, based on social comparison feedback, it might be more difficult to predict whether one’s own performance is adequate or not. Along these lines, studies investigating ambiguous feedback reported that feedback ERPs were distinctively sensitive to this manipulation (Gibbons et al., 2016; Pfabigan et al., 2015b). Thus, the observed differences between social and nonsocial reference trials also might be explainable by more low-level/non-social feedback characteristics.

In summary, we observed consistent reference frame effects during several stages of feedback processing as well as an association between internal control beliefs and FRN amplitude variation for social negative feedback in our male sample. To test whether the current results are generalizable, we conducted a follow-up experiment testing only women with the same experimental set-up.

## Experiment 2

The experimental set-up and analyses procedures were identical to Experiment 1.

### Methods

#### Participants

Thirty-six, right-handed, female volunteers (mean age 25.6 years, SD = 5.17, range 18-39) took part in the second EEG study (Oldfield, 1971). All participants had normal or corrected-to-normal vision and reported no past or current psychiatric or neurological disorder. Written, informed consent was obtained before the experiment, and it conformed to the same ethical guidelines and approval as Experiment 1. Participants performed an additional experimental task before the current one, which was not relevant for the present one and will be reported elsewhere.

#### Data analysis

Experimental procedures, EEG data preprocessing, and analysis steps were identical to Experiment 1. On average, 2.6 ICA components (range 1-5) were removed in Experiment 2. Again, artefact-free segments were averaged participant- and condition-wise—on average 42.11 (*SD* = 5.43) trials per condition.

## Results

Tables 1 and 2 summarize mean and standard deviations of behavioural, rating, and questionnaire data, Figure 2B depicts ERP amplitude courses, and Table 3 summarizes ERP descriptives. Corroborating task validity, trial-to-trial changes in response times were affected by *feedback valence* (F(1,35) = 205.21, *p* < 0.001, *η*_*p*_^*2*^ = 0.85) and *estimation adjustment* (F(1,35) = 102.88, *p* < 0.001, *η*_*p*_^*2*^ = 0.75), and their interaction (F(1,35) = 98.33, *p* < 0.001, *η*_*p*_^*2*^ = 0.74). Moreover, the three-way interaction *reference frame* x *feedback valence* x *estimation adjustment* reached significance (F(1,35) = 4.22, *p* = 0.048, *η*_*p*_^*2*^ = 0.11), and no other effects were significant (all *p* > 0.522). A Tukey post-hoc test for the two-way interaction showed that trial-to-trial changes in response times were smallest after correct adjustments following positive feedback (all *p* < 0.001) and largest after negative feedback (all *p* < 0.001). Trial-to-trial changes were comparable for correct and incorrect adjustments following negative feedback (*p* = 0.422), while they were larger for incorrect than correct adjustments following positive feedback (*p* < 0.001). The three-way interaction was driven by the observation that trial-to-trial changes were almost identical for correct and incorrect adjustments following social negative feedback (*p* = 0.999), while they were descriptively larger for correct than incorrect adjustments following nonsocial negative feedback (*p* = 0.109). The absolute difference of the difference ([negative incorrect – negative correct] – [positive incorrect – positive correct]) was larger for nonsocial (M = 109.83 ms, SD = 76.27) than social feedback (M = 82.40 ms, SD = 64.47; *p* = 0.048).

Compared with the social reference condition, the nonsocial condition yielded higher contribution to positive feedback (t(35) = −3.07, *p* = 0.004, d = 0.51) and experienced distress ratings (t(35) = −3.20, *p* = 0.003, d = 0.34). Participants felt unhappier after negative feedback for non-social than social reference trials (t(35) = 2.62, *p* = 0.013, d = 0.44). The other ratings did not differ significantly for the two reference frames (all *p* > 0.275).

The FRN ANOVA showed a significant main effect of *feedback valence* (F(1,35) = 32.61, *p* < 0.001, *η*_*p*_^*2*^ = 0.48), with larger FRN amplitudes for negative than positive feedback. The factor *reference frame* (F(1,35) = 1.35, *p* = 0.253) and the two-way interaction *reference frame x feedback valence* (F(1,35) = 1.30, *p* = 0.262) were not significant. The correlations between social negative trials and FKK-I scores (r_s_ = 0.238, *p* = 0.163) and nonsocial negative trials and FKK-I scores (r_s_ = 0.129, *p* = 0.452) were not significant. Positive feedback was not associated with FKK-I scores either (both *p* > 0.488).

The P2 ANOVA showed a significant main effect of *feedback valence* (F(1,35) = 10.93, *p* = 0.002, *η*_*p*_^*2*^ = 0.24), with larger P2 amplitudes for positive than negative feedback. The factor *reference frame* (F(1,35) = 2.82, *p* = 0.102) and the two-way interaction were not significant (F(1,35) = 1.00, *p* = 0.324). P2 amplitudes were not correlated with FKK-I scores (all *p* > 0.186).

The P300 ANOVA also showed a significant main effect of *feedback valence* (F(1,35) = 15.91, *p* < 0.001, *η*_*p*_^*2*^ = 0.31), with larger P300 amplitudes for positive than negative feedback. The factor *reference frame* (F(1,35) = 0.02, *p* = 0.890) and the two-way interaction were not significant (F(1,35) = 0.62, p=0.436). A significant negative correlation was observed for nonsocial positive trials and FKK-I scores (r_s_ = −0.350, *p* = 0.036). The other correlations did not reach significance but had the same negative associations (all *p* > 0.092). More pronounced internal control beliefs were associated with less pronounced P300 amplitudes for positive nonsocial reference frame feedback.

## Discussion Experiment 2

Experiment 2 showed no significant effects of the reference frame manipulation on the neural level. The observed feedback valence effects on all ERPs are in line with previous time estimation task results (Miltner et al., 1997; Pfabigan et al., 2015a, 2017), and serve as a quality check for task implementation in Experiment 2. However, trial-to-trial changes in response times were modulated by the reference frame manipulation. Looking at the absolute difference values suggests that participants showed larger changes in adaptive behaviour in the nonsocial than the social reference frame condition. This finding is corroborated by postexperimental subjective ratings, which were also influenced by our reference frame manipulation. Enhanced contribution and experienced distress ratings for non-social compared to social reference trials suggest a post-hoc interpretation bias in favour of the non-social condition, which showed the same direction as the trial-to-trial response time changes. Along these lines, participants rated to feel unhappier after negative feedback in the nonsocial compared to the social reference frame. The response time changes as well as postexperimental reference frame assessments suggest a slightly different effect of the reference frame manipulation in Experiment 2 than 1. Heightened generalized internal control beliefs were associated with less elaborate processing for nonsocial positive feedback, suggesting that these feedback stimuli were less important for task performance. However, the observed association between P300 amplitudes and internal control beliefs was not specific for an experimental condition, which limits its interpretation.

The absence of any significant reference frame effects on the neural level in Experiment 2 was unexpected. Of note, descriptive statistics of the three ERP components suggested an opposite pattern of the reference frame manipulation in the two experiments. Thus, we ran our analyses again with all participants, adding *experiment* as between-subject factor to explore whether the reference frame manipulation led to diverging results pattern in our two samples.

## Comparison Experiments 1 and 2

### Methods

First, questionnaire data and age were checked for potential differences using independent sample *t* tests per scale. Second, we added the factor *experiment* to the ANOVAs of trial-to-trial changes and subjective ratings. To account for increasing type I error rates inherent to repeated testing, we used a corrected *p* < 0.025. Third, to extend our initial behavioural analysis, we extracted several additional measures. We calculated the average interval length per *reference frame* and *feedback valence* as a correlate of time estimation quality. We assessed the ratio between over- and under-estimations per context (i.e., the number of reaction times longer than 1,000 ms divided by the number of reaction times shorter than 1,000 ms). Moreover, we assessed the absolute distance of each time estimation towards the 1,000 ms goal as a function of reference frame, feedback valence and estimation adjustment. We further checked whether variation in these estimations (assessed via their standard deviations) differed across conditions. The factor *experiment* was added to these analyses. Higher-order interactions including the factor *experiment* were analysed with planned comparisons in this regard. Again, a winsorizing procedure was applied per experiment and condition to the dependent variables (Wilcox, 2012). The significance level was set to *p*<0.05 henceforth. Fourth, to test the association between the repeated factors *reference frame* and *feedback valence* with *experiment* and internal control belief scores (*FKK-I),* we conducted multilevel modelling implemented in the linear mixed models module of SPSS. We chose multilevel modelling because the slopes of the covariate and the four conditions might differ from each other (Kogler et al., 2017) and a classical ANCOVA was not feasible due to almost significant difference regarding the covariate (Miller & Chapman, 2001). P2, FRN, and P300 amplitudes were modeled as a function of *reference frame*, *feedback valence, and experiment* as fixed effects and standardized *FKK-I* scores as covariate (effect-coded variables: social reference frame: 1; nonsocial reference frame: -1; negative: 1, positive: -1; Experiment 1: 1; Experiment 2: -1). A 3-level multilevel model was used accounting for *reference frame* and *feedback valence* nested within *experiment* and participants by estimating a random intercept and a random slope for each participant. We used an unstructured covariance matrix, maximum likelihood estimation, and the Satterthwaite method for estimating degrees of freedom. Semi-partial R^2^ (Edwards et al., 2008) is reported as effect size estimate for significant results; values of 0.02, 0.13, and 0.26 denote small, medium, and large effects (Cohen, 1992). Significant interactions were resolved with simple slope analysis (Aiken & West, 1991).

### Results

Demographic data: FKK-I scores were by trend higher in Experiment 1 than Experiment 2 (t(68) = 1.95, *p* = 0.055, d = 0.47; women: M = 33.08, SD = 4.31; men: M = 35.03, SD = 4.31). No differences were observed for the other FKK scales, the BSI, or participants’ age (all *p* > 0.361; Table 1).

Trial-to-trial changes in response times and subjective ratings: Trial-to-trial changes in response times showed a significant interaction of *reference frame* x *feedback valence* x *estimation adjustment* x *experiment* (F(1,68) = 5.43, *p* = 0.023, η_p_^2^ = 0.07). Using again the difference values of feedback valence and estimation adjustment as dependent variables, planned comparisons showed that significant differences between both experiments were observed for the social reference frame condition (*p* = 0.040), whereas no such differences emerged for the non-social condition (*p* = 0.616). The absolute difference of trial-to-trial changes during social reference trials was larger in Experiment 1 (M = 116.06, SD = 70.04) than in Experiment 2 (M = 82.40, SD = 64.47). In line with the results of Experiment 1, participants reported by trend to have paid more attention to feedback during the non-social than the social reference condition (F(1,68) = 5.22, *p* = 0.026, *η*_*p*_^*2*^ = 0.07). In line with Experiment 2, participants attributed higher individual contribution when receiving positive feedback to the non-social than the social reference condition (F(1,68) = 10.49, *p* = 0.002, *η*_*p*_^*2*^ = 0.13) and reported to feel unhappier after negative feedback for nonsocial than social reference trials (F(1,68) = 8.48, *p* = 0.005; *η*_*p*_^*2*^ = 0.11).

Additional behavioural analyses: The average interval length for correct estimations was influenced by *feedback valence* (F(1,68) = 239.83, *p* < 0.001, *η*_*p*_^*2*^ = 0.77) and an interaction of *reference frame* x *feedback valence* x *experiment* (F(1,68) = 4.86, *p* = 0.031, *η*_*p*_^*2*^ = 0.07). Interval length was shorter for negative than positive feedback. No other effects were significant (all *p* > 0.286). Planned comparisons for the factor *experiment* showed that the absolute difference between positive and negative feedback for social reference feedback was by trend larger in Experiment 1 (M = 11.63 ms, SD = 6.10) than Experiment 2 (M = 7.59 ms, SD = 10.90; *p* = 0.062), while no differences were observed for non-social reference feedback (*p* = 0.246). The ANOVA testing arcsine transformed ratios of the number of over- vs. under-estimations did not show any significant results (all *p* > 0.512). The absolute distance of each time estimation towards the 1,000-ms goal was affected by *feedback valence* (F(1,68) = 466.94, *p* < 0.001, *η*_*p*_^*2*^ = 0.87) and *estimation adjustment* (F(1,68) = 788.71, *p* < 0.001, *η*_*p*_^*2*^ = 0.92) and their interaction (F(1,68) = 233.13, *p* < 0.001, *η*_*p*_^*2*^ = 0.77). No other effects were significant (all *p* > 0.253). A Tukey post-hoc test showed that reaction times were farthest away from the 1,000-ms goal following negative feedback leading to incorrect adjustments (all *p* < 0.001), while they were closest to the 1,000-ms goal following positive feedback leading to a correct adjustment (all *p* < 0.001). The ANOVA on the variation of these estimations mirrored the results. Main effects of *feedback valence* (F(1,68) = 308.00, *p* < 0.001, *η*_*p*_^*2*^ = 0.82) and *estimation adjustment* (F(1,68) = 598.87, *p* < 0.001, *η*_*p*_^*2*^ = 0.90) and their interaction (F(1,68) = 45.70, *p* < 0.001, *η*_*p*_^*2*^ = 0.40) were observed. No other effects were significant (all *p* > 0.309). Tukey post-hoc tests showed largest variation following negative feedback leading to incorrect adjustments (all *p* < 0.001), but smallest variation following positive feedback leading to a correct adjustment (all *p* < 0.001).

**Figure 3.**
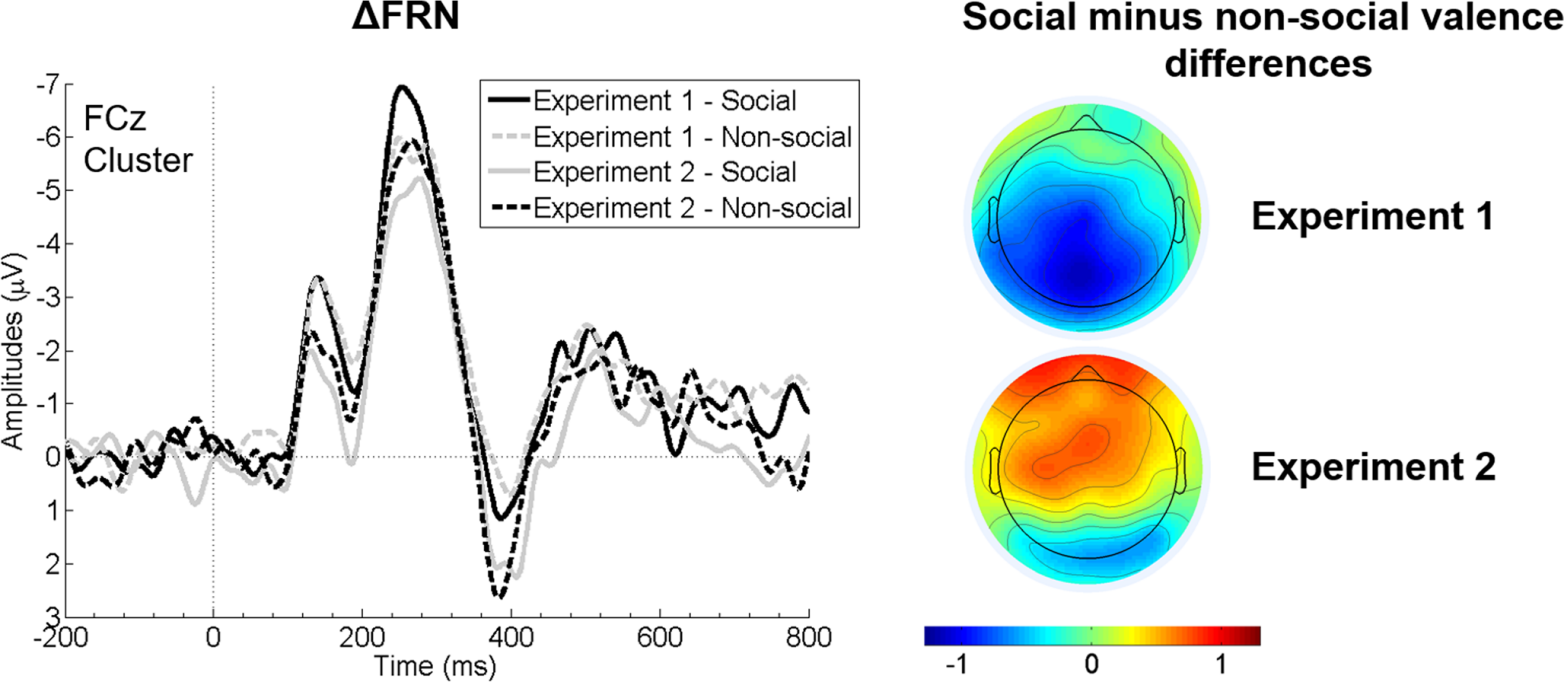
Grand average waveforms of ΔFRN (negative minus positive conditions) separately for social and nonsocial reference trials of Experiment 1 and 2 at the frontal cluster around FCz (left panel). The right-hand panel depicts scalp topographies (in μV) of the difference between social and non-social ΔFRN activation in the time window 240-300 ms after feedback onset.

**Figure 4.**
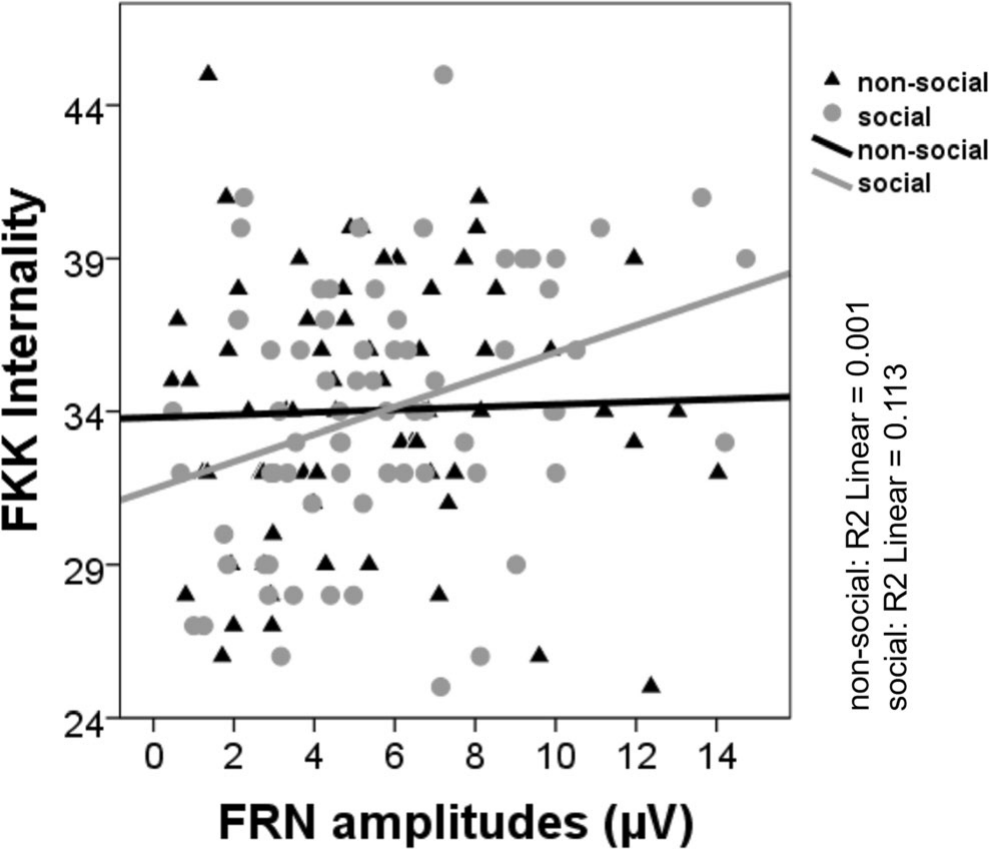
Scatter plot including regression lines of the correlation between FRN amplitudes for negative social and negative nonsocial reference feedback and generalized internal control belief sores (scale FKK-I).

Multilevel modelling: The FRN model resulted in a significant effect of *feedback valence* (b = 1.561, SE = 0.17, t(70.0) = 9.20, *p* < 0.001, semipartial R^2^ = 0.55) with larger FRN amplitudes for negative feedback. The covariate *FKK-I* moderated the effect of *reference frame* on FRN amplitudes (b = 0.256, SE = 0.09, t(70.0) = 2.727, *p* = 0.008, semipartial R^2^ = 0.10). Simple slopes analyses showed a significant positive association of FRN amplitudes and FKK-I scores in social reference trials (b = 0.629, SE = 0.28, t(157.7) = 2.28, *p* = 0.024, semipartial R^2^ = 0.03) but no association for nonsocial trials (*p* = 0.901).^1^ By trend, *reference frame* moderated the effect of *experiment* on FRN amplitudes (b = 0.185, SE = 0.09, t(70.0) = 1.99, *p* = 0.051, semipartial R^2^ = 0.05). Also, the interaction *reference frame* x *feedback valence* moderated the effect of *experiment* on FRN amplitudes (b = 0.214, SE = 0.09, t(70.0) = −2.27, *p* = 0.026, semipartial R^2^ = 0.04). To resolve this three-way interaction, we calculated ΔFRN (negative minus positive trials per condition) and conducted simple slopes analyses for the *reference frame* x *experiment* interaction. Analogue to the classical ANOVA analysis, they showed a significant positive association of FRN amplitudes and *reference frame* in Experiment 1 (b = 0.697, SE = 0.27, t(70.0) = 2.58, *p* = 0.012, semipartial R^2^ = 0.09; i.e., larger FRN amplitudes for social than nonsocial trials). In Experiment 2, a nonsignificant negative association with *reference frame* was observed (b = −0.301, SE = 0.26, t(70.0) = −1.15, *p* = 0.255; i.e., descriptively larger FRN amplitudes for nonsocial than social trials). Furthermore, they showed a significant positive association of FRN amplitudes and *experiment* for social reference trials (b = 0.783, SE = 0.39, t(109.9) = 2.03, *p* = 0.045, semi-partial R^2^ = 0.04; i.e., larger FRN amplitudes in Experiment 1 than 2), but a nonsignificant negative association for nonsocial trials (b = −0.215, SE = 0.39, t(109.8) = −0.558, *p* = 0.578). No other effects were observed (all *p* values > 0.116). As for Experiment 1, a significant positive correlation was observed for social negative trials and FKK-I scores in all participants (r_s_ = 0.328, *p* = 0.006), whereas nonsocial negative trials were not associated with generalized internal control beliefs (r_s_ = 0.115, *p* = 0.341). Steiger’s Z test showed that these two correlation coefficients differed significantly from each other (z = 2.00, *p* = 0.046; Figure 4). Importantly, the correlation coefficients of the experiment-specific correlations (men: r_s_ = 0.394; women: r_s_ = 0.238) did not differ from each other (z = 0.70, *p* = 0.484), suggesting the same direction of effect in both groups. See Figures 3 and 4 for graphical depictions of the effects.

The P2 model resulted in a significant effect of *feedback valence* (b = −1.338, SE = 0.22, t(70.0) = −6.04, *p* < 0.001, semipartial R^2^=0.34). P2 amplitudes were larger for positive feedback. Additionally, *reference frame* moderated the effect of *experiment* on P2 amplitudes (b = 0.603, SE = 0.19, t(70.0) = 3.22, *p* = 0.002, semipartial R^2^ = 0.13). Analogue to the classical ANOVA analysis, simple slopes analysis showed a significant positive association of P2 amplitudes with *reference frame* in Experiment 1 (b = 0.712, SE = 0.29, t(210.0) = 2.46, *p* = 0.015, semipartial R^2^ = 0.03; i.e., larger P2 amplitudes for social than nonsocial trials) but a nonsignificant negative association in Experiment 2 (b = −0.464, SE = 0.28, t(210.0) = −1.65, *p* = 0.101; i.e., descriptively larger P2 amplitudes for non-social than social trials). By trend, we further observed a negative association with *experiment* for nonsocial trials (b = −1.064, SE = 0.53, t(94.7) = −1.89, *p* = 0.062, semipartial R^2^ = 0.04; i.e., by trend larger P2 amplitudes for nonsocial trials in Experiment 2 than 1), whereas no significant effects of *experiment* were found for social trials (b = 0.172, SE = 0.53, t(94.7) = 0.32, *p* = 0.747).

The P300 model resulted in a significant effect of *feedback valence* (b = −1.150, SE = 0.18, t(70.0) = −6.34, *p* < 0.001, semipartial R^2^ = 0.37). P300 amplitudes were larger for positive feedback. No other effects were significant (all *p* > 0.120). In contrast to Experiment 2, no significant correlations were found between P300 amplitudes and FKK-I scores (all *p* > 0.064). The correlation coefficients of the experiment-specific correlation between internal control belief scores and P300 amplitudes after non-social positive feedback (men: r_s_ = 0.165; women: r_s_ = −0.350) differed significantly from each other though (z = −2.13, *p* = 0.032).

## General Discussion

The current study was designed to investigate the impact of reference frame (social vs. nonsocial) on performance feedback in a time estimation task, as well as the potential contribution of dispositional attribution styles in two experiments. Because our first experiment included only male participants, we repeated it with a female sample in Experiment 2. This enabled us to explore generalizability as well as potential sex/gender differences regarding the reference frame manipulation, albeit we had no specific hypotheses at the outset of the study. Postexperimental questions tapping into self-reported affect showed some differences in how participants dealt with feedback during the two reference frames. Subtle behavioural differences were observed between both experiments concerning social reference trials. On the neural level, P2 and FRN amplitudes were sensitive to the reference frame manipulation most prominently in Experiment 1. P2 amplitudes were specifically enhanced in male participants during social compared to nonsocial reference feedback. By trend, the reversed P2 pattern was observed for female participants in additional analyses. FRN amplitudes showed larger prediction error signals during social compared with nonsocial reference trials in male participants. Female participants in Experiment 2 showed weaker effects in the opposite direction, with descriptively more pronounced FRN amplitudes for nonsocial than social trials. Apart from these experiment-specific effects, generalized internal control beliefs were positively associated with FRN amplitudes in negative social compared with negative nonsocial reference feedback in all participants. In contrast, P300 amplitudes were not susceptible to the reference frame manipulation when including all participants in the statistical model.

Embedding the current results in theories on cingulate functioning, they should be considered as a further example demonstrating top-down influence of social context factors on early activation patterns, as highlighted by Koban and Pourtois (2014). These authors suggested two distinct processing hubs aiming at explaining effects of social and affective context factors during performance monitoring. The first was identified as the dorsal medial prefrontal cortex (dMPFC)/aMCC involved in fast and often automatic detection of errors, response conflict or reward prediction error signals. In line with this, FRN amplitude variation observed in the current study—most likely partly generated within these areas (Debener et al., 2005; Holroyd & Coles, 2002; Miltner et al., 1997)—corresponds to activation of this processing hub. The anterior insula was proposed as a second processing hub involved in more elaborate stages of performance monitoring such as integration of outcomes, agency, and context information (Koban & Pourtois, 2014), as well as of social affective processes (Lamm & Singer, 2010). The current study is, however, not able to provide insights regarding this processing hub. One could further consider the model of Koban and Pourtois (2014) and the current results in light of more general dual-stage models of social cognition, such as the notion of the X and C framework (Lieberman, 2007; Lieberman et al., 2002). The X-system can be considered as rather automatic and reflexive social cognition system, whereas the C-system corresponds to a controlled and reflective system. Among other brain regions, activation of the dorsal ACC/aMCC is suggested to reflect X-system activation. Thus, FRN amplitude variation could be interpreted as correlate of the X-system. Referring to this dual-stage model, Seidel et al. (2010) suggested that internal attributions might rely more strongly on automatic processes reflected in X-system activation than external attributions. This suggestion complements the observed association between dispositional internal attribution style and FRN amplitudes as indicator of early and automatic stimulus evaluation (Hajcak et al., 2006), observed in the current study as well by others (Aarts & Pourtois, 2012).

The observation that only male participants showed enhanced P2 and FRN amplitudes in response to the social reference frame condition was surprising. Previous studies addressing sex/gender differences during performance monitoring reported mixed results: enhanced amplitudes in men were observed (Clayson et al., 2011; Fischer et al., 2016; Larson et al., 2011; Yi et al., 2012) but also in women (Santesso et al., 2011; van der Veen et al., 2016). The study by van der Veen et al. (2016) even investigated social evaluation and observed enhanced FRN amplitudes in women but P300 amplitude variation only in men. It might be possible that our results stem from more general sex/gender differences in the perception of time and time estimation. As summarized by Hancock and Rausch (2010) and demonstrated in their study, women tend to underestimate short durations (such as the 1-second interval used in the current study) and show more variation in time perception than men. Indeed, we observed subtle performance differences between women and men in concert with the reference frame manipulation but no differences regarding variation in estimation behaviour in both experiments. Although the adaptive nature of Miltner’s time estimation task (1997) should take care of these between-subject differences since feedback was adjusted based on individual performance, it might still be possible that men experienced a larger discrepancy between expected and actual feedback because of their rather stable time perception ability. This discrepancy was subsequently reflected in enhanced FRN amplitudes, in particular in the social comparison context. Relatedly, a recent study reported that feedback validity was reflected in FRN amplitude variation, with low validity resulting in less pronounced or even absent prediction error signals (Ernst & Steinhauser, 2015). This might be again linked to enhanced feedback ambiguity inherent to social comparisons. Another possibility to explain the current findings is the assumption that social comparison processes are linked to some form of competition. Meta-analytic evidence of Western participants suggests that men are more willing to enter competition and competitive settings than women (Niederle & Vesterlund, 2011). Our male participants might have experienced the social reference manipulation as more competition-based than our female participants, which could have resulted in the observed P2 and FRN effects. However, because we assessed neither trait nor state competitiveness in our participants, this interpretation is speculative. Future studies are needed to clarify the possible contribution of participant sex/gender on correlates of performance monitoring—in particular when introducing social evaluation/comparison settings.

Generalized internal control beliefs were by trend larger in the male sample of Experiment 1 than in the female sample of Experiment 2. Although the association between social negative FRN amplitudes and internal control beliefs was larger in male than female participants, it was not specific for men, because the correlation coefficients did not differ between the two participant groups. Moreover, multilevel modelling corroborated the general effect of internal control beliefs over both experiments. Consequently, we assume that our initial interpretation can be applied to all participants. Those individuals scoring higher on internal control beliefs might have tried to compensate the performance uncertainty induced by the social reference frame by an increase in performance monitoring (reflected in enhanced FRN amplitudes after negative feedback). In contrast, the correlation between non-social positive P300 amplitudes and internal control beliefs was not found in all participants since its association pattern differed between Experiments 1 and 2. Of note, this correlation was not condition-specific and multilevel modelling did not find a significant association between P300 amplitudes and internal control beliefs over both experiments. Thus, we refrain from further interpretation in this regard. At last, P2 amplitudes were not associated with internal control beliefs at all. In summary, it seems unlikely that the diverging effects of both experiments might be solely explainable by generalized internal control belief scores.

However, it is possible that the observed processing differences between the two experiments were driven by other trait variables, which might interact with participants’ sex/gender. Individual preferences to engage in social comparison processes might be such a personality trait. Variation in so-called social comparison orientation (Gibbons & Buunk, 1999) was reported to account for affective evaluation of social comparison situations in a work setting (Buunk et al., 2005). High-scoring individuals reported relatively more positive affect after being better off than others but more negative affect after being worse off than others. Thus, this disposition might render social feedback more salient than nonsocial feedback for high-scoring individuals (Gong & Sanfey, 2017), which could result in enhanced feedback ERPs. Moreover, uncertainty/ambiguity of the performance standard of the social comparison group might yield processing differences in individuals with varying degrees of neuroticism and intolerance of uncertainty as shown by previous studies (Hirsh & Inzlicht, 2008; Nelson et al., 2016). Consequently, future studies should take these individual differences into consideration when planning to investigate social comparison processes.

Relating the current results to our daily lives, there is an ongoing controversy in education regarding norm-referenced (social reference) versus criterion-referenced (nonsocial reference) assessment of students’ performance (Lederman & Burnstein, 2006); the latter fosters individual learning, whereas the first emphasizes competitive behaviour. Moreover, norm-referenced assessment might lead to less optimal skill acquisition in cases were others’ behaviour is used as reference without reflection. Neuroscience research, such as the present study, can further inform our understanding of the underlying processes and help to improve educational approaches. Indeed, one study assessed neuronal activation patterns of both assessment frames during a perceptual judgement task (Kim et al., 2010) and observed diverging neuronal activation in response to both reference frame and a competence manipulation. Also, research in work psychology is interested in performance reference frames, addressing situations in which employees receive feedback from their supervisors (e.g., Zingoni & Byron, 2017). This further demonstrates the complexity of social context influences on performance monitoring and their impact on our daily lives.

It must be noted that the current reference frame operationalization refrained from explicitly introducing a competitive situation (i.e., instructing participants to perform better than the reference group). This paradigm feature may constitute a difference to educational contexts, in which individuals are often urged to perform better than others. Future studies should therefore investigate whether explicitly encouraging participants to perform better than the reference group results in comparable effects as the current study or whether this might even enhance reference frame effects.

A limitation of the current study pertains to the fact that female and male participants were tested in separate experiments. The observed sex/gender effects await testing in mixed sex/gender samples, further investigating the impact of performance reference frames. Although initially not in the focus of the current study, we believe that including the factor sex/gender provided a more comprehensive account of our experimental manipulation. Not reporting null results of sex/gender biases our perception of potential effects (e.g., see Eliot, 2011). Future studies should thus be encouraged to always report sex/gender effects if sample size allows reliable analyses.

In conclusion, early neural correlates of feedback processing were susceptible to changing performance reference frames in concert with internal control beliefs. These state versus trait influences on basic cognitive functioning translate to our daily lives as performance reference frames are applied in various contexts, such as performance evaluation of students, work evaluation of employees, or peer-review in scientific research.

## Footnotes

1 The Spearman correlation coefficient between FKK-I scores and mean FRN amplitudes for social reference trials was significant (r_s_ = 0.277, *p* = 0.020). No significance was found for non-social trials (r_s_ = 0.102, *p* = 0.401). These two correlation coefficients differed by trend from each other (z = −1.84, *p* = 0.066).
